# Paretic Dementia; Discussion before the Medico-Legal Society, of Chicago

**Published:** 1890-02

**Authors:** 


For Daniel's Texas Medical Journal.

### DISCUSSION OF PARETIC DEMENTIA.

[Stated Meeting of the Chicago Medico-Legal Society, December 7, 1889, President Dr. E. J. Doering in the chair.]

PROF. D. R. BROWER: A patient of mine was making a call in the neighborhood, and made, what I and most persons would regard as a very indecent proposal to a lady who was a friend of his wife. He committed other extravagances of that sort, until it became necessary to have him adjudged insane, and three distinguished experts in the State of New York sat upon his case. These three men are eminent in the profession and especially eminent in the psychological department of medicine. One of the three was satisfied that he had general paralysis of the insane; a second said that he did not have it, that the symptoms he showed were simply the effects of alcohol; and the third, that he might be insane, but not sufficiently so to warrant his incarceration; and the result was that he came through that ordeal with flying colors, and then we were all to be sadly punished as soon as he got back to Chicago. He took a steamer and went to Europe, and while there behaved himself with great decorum. I have recently seen a gentleman who traveled with him in Europe, and he told me that he was as perfectly himself, seemingly, as he ever knew him to be, and he would not believe the stories he had heard. He returned from Europe and acted as if he were going to be himself again. It seemed as though those who had supposed that he had general paralysis, had been mistaken. Soon he began to be very melancholy and talked about suicide, and this condition continued until his friends again became alarmed about him, and he was then placed in an insane hospital. Since his incarceration in the hospital he has again taken upon himself these grand illusions of the same general character, just as he had when he filled his house with the most elegant things, bought costly paintings and choice bric-a-brac, and the finest furniture, and made several large real estate transactions—immense transactions for a man of his financial capacity. This is a case of general paresis; there is no doubt about the diagnosis now, he has all the symptoms clearly defined. He has the exalted, grand delusion, the motor disturbances that accompany the disease, the trembling of muscles, the thickness of speech, the uncertainty in the locomotion, the pupillary phenomena, the unequally dilated pupils, the want of pupillary response to the light; he has now emotinal and intellectual weakness; he has now, clearly defined, all the symptoms that are characteristic of general paresis.

The case began in headaches, irritability, then came these grand delusions, which subsided, and he seemed to be perfectly well, and when this period of subsidence came on he deceived two of the very best experts that I know of. And then there was a little period when there was depression; then there came on again these symptoms of exaltation, these paretic symptoms, this emotional and intellectual weakness. Some cases of general paralysis are not so difficult as this; there is no remission worth speaking of; there is nothing but a gradual progressive increase in all the symptoms. But there is another class of general paretics that are a source of great annoyance occasionally. I mean those cases of general paresis that begin with mental depression, the type of mind in the general paretic being exaltation. I think it is Hamilton who speaks of two cases he had in the hospital in New York; one owned all the ships in the world, and the other owned all the grain in the world, and he found them one day bargaining with each other, the man who had the grain wanted the ships to export it. In this exalted type of manifestation they have all the wealth that is possible. I have a general paretic under observation who owns all the flour in the market. I asked him to-day if he would not let me have a barrel for my own use, and he said "No, not a barrel; I can't spare it."

While this is the usual type of general paresis, occasionally we meet with general depression; and then we are often much perplexed in the diagnosis, but fortunately the cases that have mental depression are not usually cases of legal interest. I think the depressed condition of mental manifestation is more apt to occur in women, and is much more frequently found in paretics who have a tubercular inheritance. Fortunately, these cases of general depression with mental paresis are but few. While the mental depression continues, we have the motor disturbances and the emotional and intellectual weakness to aid us in the diagnosis. I think it is very uncommon for this depression to last very long; it usually comes on in the beginning, and lasts a few months, and then gradually gives place to these expansive ideas, these delusions of grandeur. When general paresis first begins, the disease, I think, very often has legal relations that pass unrecognized—the relation between the criminal act and the general depression, and every now and then you will find something similar to what I saw in the papers a few weeks ago, as occurring in the central part of the State. A gentleman who was at the head of a female seminary, and had spent some twenty years in the education of girls, and had been most successful, was accused of making an indecent proposal to one of his scholars. For twenty years this man had led an examplary life; this was the first offense, and, of course, it broke up the school and a great commotion was caused in the neighborhood. That case, I believe, will turn out to be a case of general paresis. I have met with several just such cases; people who all their lives, up to forty-five or fifty years have been perfectly exemplary in all their habits, all of a sudden get into these habits of extravagance, irritability and lasciviousness. Such cases are not uncommonly general paresis, and if you study them closely, notice the trembling of the muscles, the peculiar character of the speech, the motor weakness, and the general tremor that accompanies that weakness, and find out the expansive delusions, then you have a case of general paresis, more or less marked.

DR. ARCHBALD CHURCH.

MR. PRESIDENT: It may be well to briefly outline some of the salient features of general paralysis of the insane, in order to have a basis on which to work. In the first place it is considered by the majority of writers as being invariably a fatal malady. Some equally authoritative writers, however, insist that there have seen cases of this form of insanity which have undoubtedly recovered, and subsequently lived a number of years and followed occupation, thus proving complete recovery, in their estimation. Those of the first class insist, however, that there must have been a mistake in the diagnosis, and that the fact that these patients lived is prima facie evidence that they could not have had general paralysis. It is a disease which has a distinct pathology, and unlike most forms of insanity when we encounter a case we know what we will find post mortem. We will find a peculiar condition of the convolutions of the cerebrum, and stripping the membranes from the brain, a surface portion of the gray matter of the gyri will follow the dura mater, so that the brain is said to be decorticated. These patients present no pupillary phenomena and peculiarities of the reflexes already pointed out by Dr. Brower. The great leading feature of their mental condition is the egotistical attitude which they assume in regard to their personal relations to life. A patient of this sort will sometimes fall into a violent rage at the merest trifle. He feels that the slightest annoyance is a matter of moment, because it is the annoyance of a man of considerable importance, whom he greatly admires. On the other hand, matters of real importance, for instance the loss of fortune, scarcely affect him because he feels that is a mere trifle for a man of his ability to lose an ordinary fortune. He will tell you that his wife is the most beautiful woman in the world, that her eyes are diamonds and her finger-nails opals, and that there is no limit to her value. So he will be a monarch, a Cæsar, a Napoleon, all at the same time. In the most foolish, rambling way, he expresses these delusions of grandeur. When a patient presents these symptoms the diagnosis is no longer a matter of difficulty; anyone can see that he is bereft of reason, and one of a little experience with insanity will at once recognize the character of his malady. But, as has been well pointed out by many, the disease really antedates all mental manifestations, sometimes by months, even by years, so that by the time the patient manifests aberration he has long been the subject of those pathological conditions which are found post mortem. This was very vividly impressed upon my mind by post mortem examinations I made of two patients who died in the early period of the so-called first stage of this disease. They had been sequestrated by due process of law, and at the time of their death had manifested mental symptoms for only about three weeks. Ordinarily people do not submit their friends to legal process at such an early period, but hope by one maneuver or another to restore them to reason.

In these two cases the patients had manifest mental trouble only for a short time before their death which was caused by the epileptiform, attacks peculiar to this disease, and from which scarcely a case is ever entirely free. In these instances the patients died in the first convulsive attack. On post mortem I found the pathological conditions I have outlined, well developed over the vertex of the brain, showing that the pathological substratum, or fonndation of the disease had been in existence at least many months. Spitzka will say that in the majority of instances where you can follow backwards the history of the patient, you will be able to determine the disease as antedating the outbreak a year or two years, and that has been my experience. It strikes me that it is of the highest importance to recognize if possible the disease in this early stage before it has reached that point where the patient becomes unable to follow any pursuit, and before his condition demands that he should be removed from contact with society. As I said, the disease is extremely fatal, and if there is any chance of recovery it must be in the early stages, and every effort should be bent towards placing these patients at the earliest moment under proper care, observation and treatment. There are certain indications which, while in themselves scarcely of sufficient importance to warrant us in saying a man will be a paertic, still will justify a suspicion of the oncoming disease, and also justify the medical man in warning the relatives of what they may expect and enable him to indicate to them the proper measures to be taken to prevent the outbursts of the disease and the possibly deplorable results of the patient's conduct. They may in this way prevent his dissipating a fortune and bringing want upon those who are dependent upon him, or from getting into some escapade which may be extremely humiliating to his friends, and possibly from doing some terrible deed. In the very earliest stage of the disease there is ordinarily a period of depression. I have been able to verify this in a few instances only, but often the very first thing you can find out by closely questioning those who have been a long time associated with the patient will be that at some time, a year or possibly two or more years before, he has undergone a period of depression, and very often this is accompanied by severe and continuous pain in the head. Its duration is variable and on the whole not of a great length of time. He recovers from it almost completely, and gradually puts on an appearance of excellent health, and expresses a great deal of self-satisfaction, not only with his present worldly state, but with his future prospects, and particularly with his physical condition. This elementary feature, if I may so call it, this period of depression, I consider of some importance. The next important feature of early appearance is the failure of the memory, not that he cannot remember items if you call them to his attention, or that he cannot recollect any important matter in which he may have been engaged, but little matters like appointments, for instance, will slip his mind while formerly he may have been punctilious in this respect; and business items which should be a matter of record will escape his attention. This a uniform symptom sooner or later in the disease, and associated with this if you can get at his letter writing you will find that he omits words necessary to the construction of the sentence or frequently repeats words unnecessarily. Later on his handwriting is characteristic, and any expert will tell you that it is the handwriting of a paretic, but at this early stage the only peculiarity in the writing will be the omission or duplication of words. If now you examine his eyes, in a number of cases, the percentage I cannot state, but is large, you will find eye symptoms of grave significance and great importance. The pupils will not be equal in their dilatation or contraction; they may be closely contracted, or the tendency may be to wide dilatation, but they will not be equal. One pupil or the other, or possibly both will present an irregularity in its outline, due to the unequal action of the dilator, analagous to the general muscular inco-ordination of a later phase. Then if you will look at his tongue in the very early stage of the disease you will notice a peculiar fibrillary twitching on the dorsal surface of the organ that is different from the tremulousness of the tongue later in the disease. It will remind you of a loose fabric—as if a looser strand were woven into the fabric and this single strand set in motion. It is as if a few of the muscular fibres of the dorsum of the tongue acted without the associated motion of their fellows. The patella reflexes are exaggerated even in the earliest stages of this disease. When you find these pupillary phenomena, with increased knee jerk, and particularly if there is hyperæmia of the retina, if the patient has a history of a depressed period associated with pain in the head, and the memory has also shown a tendency to fail, and if the patient has been loose in his manner of living, all these things will point to the probability of the oncoming disease. Though they may not justify a diagnosis or a prognosis they will justify the medical man in warning the friends and relatives of the critical condition in which the man stands and enable them to take some precautions and place about him some safeguards in regard to the disposition of his property, and possibly to protect him from those outbreaks which are characteristic of a later stage of the disease, and which often entail grave consequences.

DR. H. N. MOYER.

MR. PRESIDENT: I am unfortunately still in a position to have little to say, because the remarks I had purposed making have been so well stated by Dr. Brower and Dr. Church. Of course I could discuss this question in *extenso* if I differed from them, but as I do not, I must necessarily be brief. Particularly, I wish to call your attention to, and emphasize one point; that is, the difficulty of making a diagnosis in some cases of paretic dementia. The early symptoms have been fully and graphically described, so that no one can fail to get a distinct idea of this dreadful disease; but, unfortunately, there are a few cases in which this disease does not present these typical features. Instead of the delusions of grandeur and the exaltations so characteristic of the disease, we may have depression during all the early stages. I think it is an almost universal experience of those in charge of our asylums, that a certain number of patients will be admitted each year who will be classed among other forms of insanity, but will turn out to be paretic dements. These cases were all mistakes in diagnosis, as paretic dementia is not a terminal form of other psychoses, but existed in these patients from the beginning of their mental troubles. This is not to deny that paretic dementia may occur as a complication in the course of other insanities, paranoia, for example, as has been pointed out by Spitzka.

During the past year, I was called to a neighboring summer resort to see a man who had suddenly manifested marked signs of insanity. I was suspicious from the start that we had to do with a case of paretic dementia, but the age of the man and the symptoms were rather against this view, so that a diagnosis was made of insanity from coarse brain disease, the patient having been hemiplegic from the beginning of the attack.

Later this man returned to the city, and I was called upon to attend him in an epileptiform convulsion, from which he emerged with paralysis upon the opposite side from that upon which it was first observed. Later he presented all the typical symptoms of paretic dementia.

The only thing that I have to add to the remarks of previous speakers, is to emphasize the fact that we have typical cases, and that it is of the utmost importance for the family physician to recognize these cases early, before they have had time to squander their property, get into jail, or disgrace their friends

A case I have in mind occurred recently in this city, where a man squandered $40,000 in a single week. He bought everything, from a wagon load of matches to a hundred thousand dollar piece of real estate. It was only after he had markedly diminished his estate that his true condition was discovered, and means taken to secure legal control of him and his property. We must always look to the family physician for the early recognition of these cases—they do not reach the specialist until later.

DR. DOERING—Does Dr. Moyer know of an instance where such matters have been brought into court; for instance, where a contract is made unfavorable to the patient?

DR. MOYER—Yes, the courts have ruled in many instances. In the case of one of the patients I mentioned, I was consulted, and the question came up as to whether the patient was competent, in the second stage of this disease, to dispose of his property. The amount at issue was large, and a prominent real estate lawyer of this city was consulted. He gave it as his opinion that the transfer would be legal, as it could be shown that it was done to conserve the estate of the person, and while he yet had sufficient mental power to appreciate the nature and quality of his act.

The courts have always held that these contracts were voidable, but not void *ab initio*. The view taken of them largely depends upon whether they are contrary to the interests of the patient, and particularly their equity would be a subject for inquiry.

DR. C. E. WEBSTER—I would like to remark that the case stated by Dr. Brower is a very suggestive one. I have often thought, when considering these questions of alienation, etc., as I have, in the general way, and once in a while I have in a very particular way, that a person who was incapable of a useful life in American society might, as Dr. Bower's patient did, by going abroad into another state or society, among other surroundings, be useful for a much longer period of time.

I do not wish to enter into a disquisition, but one of my Scandinavian acquaintances has told me, and his statements are to be depended upon, that the method that is adopted in some European countries, in a general way, is of placing the patient in a different phase of life. This is carried out in Sweden to his knowledge, by a certain man of the clerical profession, who takes demented people and goes out with them into the fields and woods. He takes them into training, and after awhile gets their minds recalled and reco-ordinated so that they live useful lives. This Swedish priest is held by the State of Sweden as a foe, and his life is constantly menaced; he has often been under arrest, but still he continues gathering together the broken fragments of the demented people and restoring them to usefulness in the way I have mentioned, by taking them out into natural surroundings and freeing them from the restraint of that life which irritated them.

